# High-speed free-run ptychography at the Australian Synchrotron

**DOI:** 10.1107/S1600577521012856

**Published:** 2022-01-17

**Authors:** Michael W. M. Jones, Grant A. van Riessen, Nicholas W. Phillips, Christoph E. Schrank, Gerard N. Hinsley, Nader Afshar, Juliane Reinhardt, Martin D. de Jonge, Cameron M. Kewish

**Affiliations:** a Central Analytical Research Facility, Queensland University of Technology, Brisbane, Queensland 4000, Australia; b Department of Chemistry and Physics, La Trobe Institute for Molecular Science, La Trobe University, Bundoora, Victoria 3086, Australia; c Melbourne Centre for Nanofabrication, Clayton, Victoria 3168, Australia; d Department of Engineering Science, University of Oxford, Parks Road, Oxford OX1 3PJ, United Kingdom; e School of Earth and Atmospheric Sciences, Faculty of Science, Queensland University of Technology, Brisbane, Queensland 4000, Australia; f Australian Nuclear Science and Technology Organisation, Australian Synchrotron, Clayton, Victoria 3168, Australia

**Keywords:** ptychography, scanning X-ray diffraction microscopy, coherent diffractive imaging, ultramicroscopy, phase-contrast imaging

## Abstract

Over the last decade ptychography has progressed rapidly from a specialist ultramicroscopy technique into a mature method accessible to non-expert users. However, to improve scientific value ptychography data must reconstruct reliably, with high image quality and at no cost to other correlative methods. Presented here is the implementation of high-speed ptychography used at the Australian Synchrotron on the XFM beamline, which includes a free-run data collection mode where dead time is eliminated and the scan time is optimized. It is shown that free-run data collection is viable for fast and high-quality ptychography by demonstrating extremely high data rate acquisition covering areas up to 352 000 µm^2^ at up to 140 µm^2^ s^−1^, with 13× spatial resolution enhancement compared with the beam size. With these improvements, ptychography at velocities up to 250 µm s^−1^ is approaching speeds compatible with fast-scanning X-ray fluorescence microscopy. The combination of these methods provides morphological context for elemental and chemical information, enabling unique scientific outcomes.

## Abstract 2

The implementation of high-speed ptychography on the Australian Synchrotron XFM beamline in presented. It includes a free-run data collection mode where dead time is eliminated and scan time is optimized. This data collection mode is compatible with fast-scanning X-ray fluorescence mapping with extremely high data acquisition rates over large areas, demonstrated at up to 140 µm^2^ s^−1^ with 13× spatial resolution enhancement compared with the beam size.

## 1. Introduction

Scanning X-ray diffraction microscopy (SXDM), a modern version of ptychography (Hegerl & Hoppe, 1970 ▸), is a high-resolution high-sensitivity scanning coherent diffractive imaging method. Phase and absorption images of the object, projections of the complex X-ray refractive index, are recovered through an iterative reconstruction algorithm (Faulkner & Rodenburg, 2004 ▸; Thibault *et al.*, 2008 ▸). In recent years, the development of SXDM has matured to deliver scientific outcomes (*e.g.* Kourousias *et al.*, 2018 ▸; Jones *et al.*, 2019 ▸; Genoud *et al.*, 2020 ▸; Schrank *et al.*, 2021 ▸), particularly in the context of 3D nanostructure analysis (*e.g.* Chen *et al.*, 2013 ▸; Trtik *et al.*, 2013 ▸; Bø Fløystad *et al.*, 2015 ▸; Ciani *et al.*, 2018 ▸). The simultaneous application of SXDM with X-ray fluorescence microscopy (XFM) (Vine *et al.*, 2012 ▸; Deng, Vine *et al.*, 2015 ▸; Jones *et al.*, 2016 ▸) provides super-resolution ultrastructural context for the fluorescence data. Key developments enabling correlated high-throughput SXDM-XFM are the development of fly-scanning SXDM data collection (Clark *et al.*, 2014 ▸; Pelz *et al.*, 2014 ▸; Huang *et al.*, 2015 ▸; Deng, Nashed *et al.*, 2015 ▸) and mixed-state reconstruction (Thibault & Menzel, 2013 ▸), which accounts for partial coherence of the beam and object motion during acquisition. These developments, *inter alia*, have allowed ptychographic imaging to be achieved with unprecedented scan areas and data collection rates. Examples include a nematode imaged in a scan area of 82 000 µm^2^ at 6.4 µm^2^ s^−1^ (Jones *et al.*, 2019 ▸), an integrated circuit imaged in optimized step-scan mode over 145 000 µm^2^ at 34.5 µm^2^ s^−1^ (Guizar-Sicairos *et al.*, 2014 ▸) and an integrated circuit imaged with a double-multilayer monochromator (DMM) over 9 µm^2^ in 1.2 s (Deng *et al.*, 2019 ▸). The advent of diffraction-limited storage rings with increased coherent flux and broadband ptychography algorithms that can exploit DMMs (Enders *et al.*, 2014 ▸; Yao *et al.*, 2021 ▸) provides opportunities (Thibault *et al.*, 2014 ▸) for further gains in data collection rates, with prospects of obtaining nanoscale images of macroscopic specimens (Du *et al.*, 2021 ▸).

X-ray microprobes are moving towards event-mode data streams. One common approach is to use a spatially delimited event stream, where image-space pixel boundaries are used to delimit the continuous arrival of a stream of fluorescence photons, to record and reset counters, *etc.* The Maia detector system (Ryan *et al.*, 2018 ▸) was one of the first to adopt this approach, collecting a stream of event-mode data and eliminating per-pixel readout overheads, facilitating XFM imaging in the megapixels-per-hour regime (Howard *et al.*, 2020 ▸). A second approach uses a rapid time-based sampling of spatial locations to delimit the stream of recorded events (Deng *et al.*, 2019 ▸). This article reports a retrofit of an SXDM measurement system onto an existing X-ray fluorescence microprobe that uses a spatially delimited event-mode triggering system.

One of the fundamental challenges of combining a spatially delimited system with a timed exposure detector, such as a pixel array detector typical for SXDM, is the mismatch of units: space converts directly to time only when velocity is constant. In essence, velocity variations can result in a race condition between the end of an SXDM exposure and the arrival of the next pixel boundary. The result is that, at best, the detector ignores the trigger received while acquiring, finishes the acquisition of the current frame and then waits until the next trigger from the subsequent scan point, resulting in a missed frame. At worst, depending on the detector electronics, the arrival of a trigger during acquisition may result in an error state, aborting the scan. To avoid this race condition, a per-pixel safety margin in the form of scan or detector dead time is introduced to ensure a trigger is not issued before the detector is ready (Jones *et al.*, 2016 ▸). Here we propose an alternative where triggering is avoided altogether by allowing the detector to acquire images continuously.

## 2. Implementation

On the Australian Synchrotron XFM beamline, we have implemented SXDM using a detector-driven free-run data collection method, where the detector internally triggers exposures according to a configured sequence. An EIGER2 X 1M (Dectris Ltd) hybrid photon-counting detector has been provided through a collaboration between La Trobe University and ANSTO. This detector retriggers with practically no (0.1 µs, <0.02%) dead time between the end of an exposure and the beginning of the next, meaning that all photons delivered to the sample contribute to the final image. The rising edge of an ‘acquisition in progress’ logic signal is recorded as a frame event into the Maia data stream, tagged with a stage event containing the current position of the sample stage 20 nm encoders. A time stamp is not required since the frame event is associated with position only. The continuous nature of the data acquisition permits estimation of the centre of the sample motion kernel during each SXDM frame exposure by linear interpolation of the stage events.

In free-run data collection mode the race condition between sample scan-point arrival time and detector framing completion is eliminated. Furthermore, the frame rate can be varied independently from the XFM pixel dwell time, allowing, for example, a significantly higher sampling rate in the fast scan direction than on the slow axis, which has been shown to be beneficial for fly-scanning SXDM (Pelz *et al.*, 2014 ▸). Here, we demonstrate free-run SXDM data collection at scan velocities up to 250 µm s^−1^ and compare the reconstructed results with fly-scanning SXDM using position-based external triggering at each scan point. We apply simultaneous free-run SXDM-XFM imaging to a freestanding thin section of a rock sample featuring the interface between shale and a quartz–calcite vein. The results show that large-area high-resolution imaging provides inherently correlated nanoscale structural context for elemental mapping, providing unique scientific insights that cannot be realized with either method independently.

## 3. Data analysis pipeline

**Figure 1:**
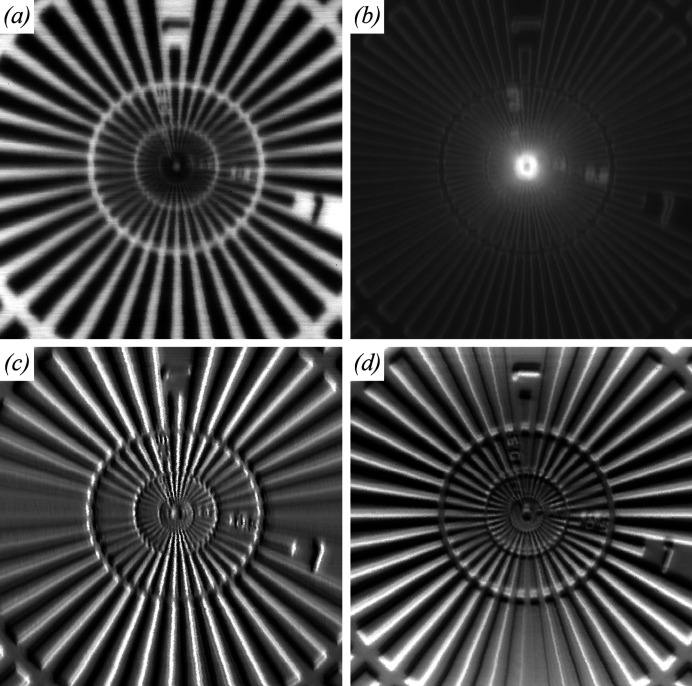
Scanning transmission X-ray microscopy (STXM) images representing (*a*) transmission, (*b*) darkfield (scattering, shown on a logarithmic scale), and (*c*) and (*d*) differential phase contrast in horizontal and vertical directions, respectively. Each 100 nm wide pixel in these 50 µm × 50 µm images is calculated from a single detector frame and the spatial resolution is limited by the beam size, here 350 nm × 550 nm in the focal plane.

A Python data analysis pipeline was implemented on the XFM beamline to streamline SXDM data processing. Detector frames are stored as bitshuffle-LZ4 (ESRF Data Analysis Unit; *hdf5plugin for Python*, https://pypi.org/project/hdf5plugin/) compressed HDF5 data in the detector control unit RAM. Data files containing *N* frames are automatically transferred to the beamline file server as the files become available. The files are sorted, based on sample identification and scan number, into an iteratively traversable folder structure. A cluster of compute nodes on the beamline, having 240 CPU cores and 2.3 TB RAM, produces near real-time scanning transmission X-ray microscopy (STXM) images (Menzel *et al.*, 2010 ▸). That is, transmission, darkfield and differential phase-contrast images (Fig. 1 ▸), which are used for immediate feedback on targeting and data quality to guide experiment progress.

The data analysis pipeline further pre-processes and packages the HDF5 data and relevant beamline metadata for SXDM reconstruction. The frame and stage events are extracted from the Maia data stream. SXDM data are corrected by a predefined valid pixel mask and the beam centre is automatically located from the sum of the frames in the first data file. Then, a region of interest (ROI) of *M* ^2^ pixels surrounding the beam centre is extracted, where *M* is usually between 128 and 512 depending upon the required reconstruction pixel size. SXDM reconstructions are then typically carried out on the beamline cluster using a mixed-states difference-map and maximum-likelihood-based approach (Thibault *et al.*, 2008 ▸; Thibault & Guizar-Sicairos, 2012 ▸; Thibault & Menzel, 2013 ▸; Enders & Thibault, 2016 ▸). However, in the present work, SXDM data were cropped to *M* ^2^ = 256^2^ pixels and reconstructed with the *ePIE* algorithm (Maiden & Rodenburg, 2009 ▸) for 2000 iterations using ten orthogonal probe modes in a parallel GPU-enabled environment (Nashed *et al.*, 2014 ▸), yielding reconstructed images with a pixel size of 24.5 nm.

## 4. Experimental method

The XFM beamline geometry has been described previously (Howard *et al.*, 2020 ▸). The double-crystal monochromator selected 10 keV radiation from the undulator spectrum. With the white-beam slits in a typical XFM configuration, opened to 1.1 mm horizontal (h) × 0.2 mm vertical (v), and the position and intensity of the beam at the secondary source aperture maintained by a PID controller monitoring feedback from a quad-diode beam-position monitor, the beam was focused to approximately 2 µm (h) × 2 µm (v) full width at half-maximum (FWHM) by the Kirkpatrick–Baez mirrors. The beam-positioning feedback was then turned off during all SXDM measurements to avoid movements of the upstream optics that may affect the probe during scanning. A coherent portion of the beam was selected by closing the white-beam slits to 0.1 mm × 0.1 mm and the secondary source aperture (SSA) was set to 0.008 mm × 0.2 mm, where the horizontal size is used to control the beam intensity to avoid saturating the detectors. This reduced the beam size at the focal plane to around 350 nm × 550 nm FWHM and the beam intensity by a factor of around 40. The Maia fluorescence detector was in its usual backscatter geometry and collected fluorescence photons, which were processed using *GeoPIXE* (Ryan & Jamieson, 1993 ▸; Ryan *et al.*, 2015 ▸). The EIGER2 SXDM detector, a fast-framing array of 1028 × 1060 75 µm wide pixels, was located 3.88 m downstream of the focus. A resolution test pattern made from two layers of 450 nm Au and 60 nm Cr was scanned downstream of the focal plane. Defocus is used to tune the beam (or probe) size on the sample. In this case the sample was scanned at 4 mm defocus where the probe was around 2 µm wide. Continuous-motion fly scanning is carried out using a raster-scanning motion sequence (*RASCAN*) which pre-calculates a bidirectional scan path to cover the scan area with optimized time and precision at the requested scan velocity and scan line separation (Afshar *et al.*, 2017 ▸). As the sample approaches the end of the scan line, the non-scanning axis begins to accelerate to the next scan line, achieving a parabolic turnaround just outside of the defined scan region, minimizing both the scan line transition time and the total scan time.

Fig. 1 ▸ shows an STXM image of the test pattern, scanned in the focal plane with 0.1 µm sampling interval over a 50 µm × 50 µm scan area, where panel (*a*) shows transmission, (*b*) shows darkfield on a logarithmic intensity scale, and (*c*) shows horizontal and (*d*) vertical differential phase contrast. The brightest areas in Fig. 1 ▸(*b*) represent the strongest scattering from features within the beam at each scan position. Overview STXM scans were obtained for targeting purposes with a 1 µm sampling interval over large areas, *e.g.* 0.5 mm × 0.5 mm, with a 1000 Hz frame rate, taking under 5 min per scan and yielding of the order of 275 000 frames. The data analysis pipeline distributes these data to the cluster and obtains STXM images from such a dataset in 1.5 min. ROIs were chosen from darkfield images for high-resolution SXDM scanning.

A baseline comparison of position-based externally triggered (ET) and free-run (FR) data collection modes was carried out. The sample was scanned in both modes with 100 nm sampling intervals in both the horizontal and vertical directions at a scan velocity of 5 µm s^−1^ at a frame rate of 50 Hz, *i.e.* 20 ms exposure per frame, yielding a data collection rate of 0.49 µm^2^ s^−1^. For ET data collection, the frame exposure time was set to 0.99 times the expected XFM dwell (pixel transit) time. This exposure time was chosen to provide a 1% safety margin to avoid the race condition described above while having approximately the same number of photons per frame as the FR scan.

Scan speed tests were carried out on a second test pattern of similar composition at 100 nm and 500 nm sampling intervals in the horizontal and vertical directions, respectively, at a horizontal velocity of 250 µm s^−1^ and a frame rate of 2500 Hz, *i.e.* 0.4 ms exposure time per frame, yielding a data collection rate of 140 µm^2^ s^−1^. The detector dynamic range reduces from 16 bits to 8 bits at frame rates above 2250 Hz, and the maximum count rate capability per pixel in 8 bit mode is reduced. The beam intensity was accordingly reduced by a factor of 10 for this scan by reducing the SSA dimensions to 0.004 mm × 0.02 mm.

To demonstrate fly-scanning free-run SXDM at high data rates correlated with XFM over a large area of a heterogeneous sample, a freestanding 30 µm thick section of shale containing a small calcite vein was scanned over an area of 800 µm × 440 µm. XFM maps were obtained at an incident energy of 10 keV, yielding 1.410 megapixel XFM images for 15 elements including K to Zn, Ce, Ba, Nd, Hf and Yb, simultaneously with a correlated 523 megapixel SXDM image when reconstructed at 24.5 nm pixel size. The sampling interval was 500 nm in the horizontal and vertical directions and the scan velocity was 150 µm s^−1^ at a frame rate of 300 Hz, *i.e.* 3.33 ms exposure time per frame, yielding a data collection rate of 74 µm^2^ s^−1^. The whole scan consisting of 1.418 million frames took 79 min. There are more SXDM frames than XFM pixels because the detector continues freely acquiring frames during the ∼30 ms turnaround between scan lines, outside the XFM scan area.

## 5. Results and discussion

**Figure 2:**
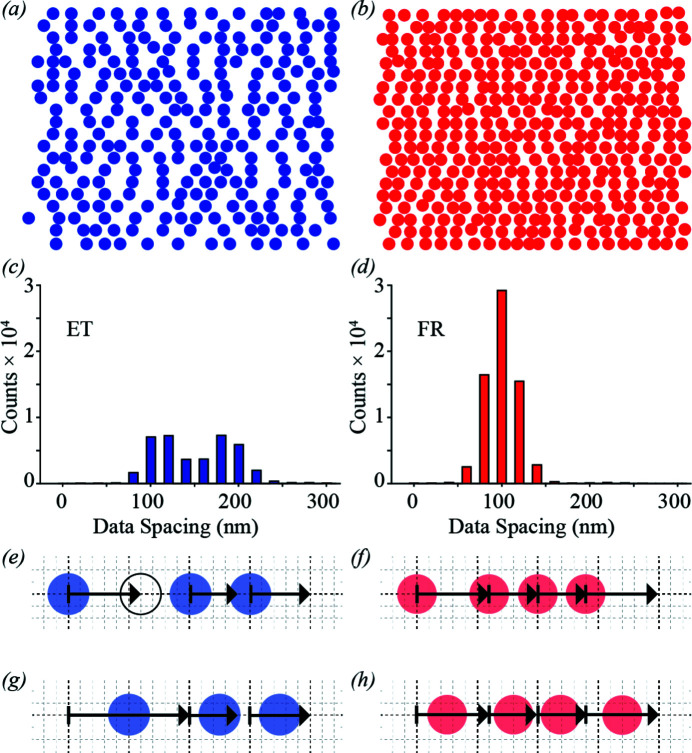
Comparison of fly-scanning SXDM positions in (*a*) externally triggered (ET) mode and (*b*) free-run (FR) mode, over a 2 µm × 2 µm scan subregion of a 50 µm × 50 µm scan. (*c*) The histogram of ET point spacing for the full scan is bimodal, while (*d*) the FR point-spacing histogram for the full scan is unimodal, see main text for discussion. (*e*) An illustration of the ET position assignment, where the heavy dashed vertical lines represent the pixel boundaries where triggers are sent to the detector, and the exposure time for each frame is indicated by the length of the arrow from the bar to the end of the arrowhead. The empty circle shows a missed frame when the trigger is issued during an exposure. (*f*) An illustration of the FR position assignment, where the light dashed lines represent the 20 nm encoder counts. (*g*) Interpolation of the positions in ET mode results in inaccurate estimation of the centre of the frames preceding a missed frame, while (*h*) in FR mode, the centres of all frames are accurately estimated. Note that the probe size is many times larger than the distance between stage events.

Interrogation of the positions where frames were acquired in each scan mode reveals the extent of missing data in ET compared with FR data collection. Figs. 2 ▸(*a*) and 2 ▸(*b*) compare the start-of-frame positions (stage events) for a 2 µm × 2 µm scan subregion of a 50 µm × 50 µm scan in ET and FR mode, respectively, and Figs. 2 ▸(*c*) and 2 ▸(*d*) show the full scan histograms of point spacings in these modes. The ET histogram is bimodal, with peaks centred at 100 nm and 200 nm having similar frequency. In contrast, the FR histogram has a single 100 nm peak [Fig. 2 ▸(*d*)], where the peak width is attributed to velocity variation of the scanning stage. Each instance of 200 nm spacing in the ET data represents a missed frame [Fig. 2 ▸(*e*)]. This is visually evident in Figs. 2 ▸(*a*) and 2 ▸(*b*) and is confirmed by comparing the sizes of the FR and ET datasets, where the latter includes only ∼66% of the number of frames compared with FR for a given scan area [Figs. 2 ▸(*e*) and 2 ▸(*f*)]. Missed frames result in an increased effective detector dead time and fewer photons being collected per unit scan area. At higher velocities, or larger sampling intervals, the missing frames can result in insufficient probe overlap for reliable SXDM reconstructions. Furthermore, it is difficult to estimate the centre of the sample motion kernel, as the end-of-frame position is unknown [Fig. 2 ▸(*g*)]. Fig. 2 ▸(*h*) illustrates position interpolation in FR mode. Since the frame start and end are known, the centre of the sample motion kernel can be estimated. On the basis of obtaining more frames per unit area with more accurate positions for each frame, it is expected that the ptychographic image quality must be better for FR than ET.

**Figure 3:**
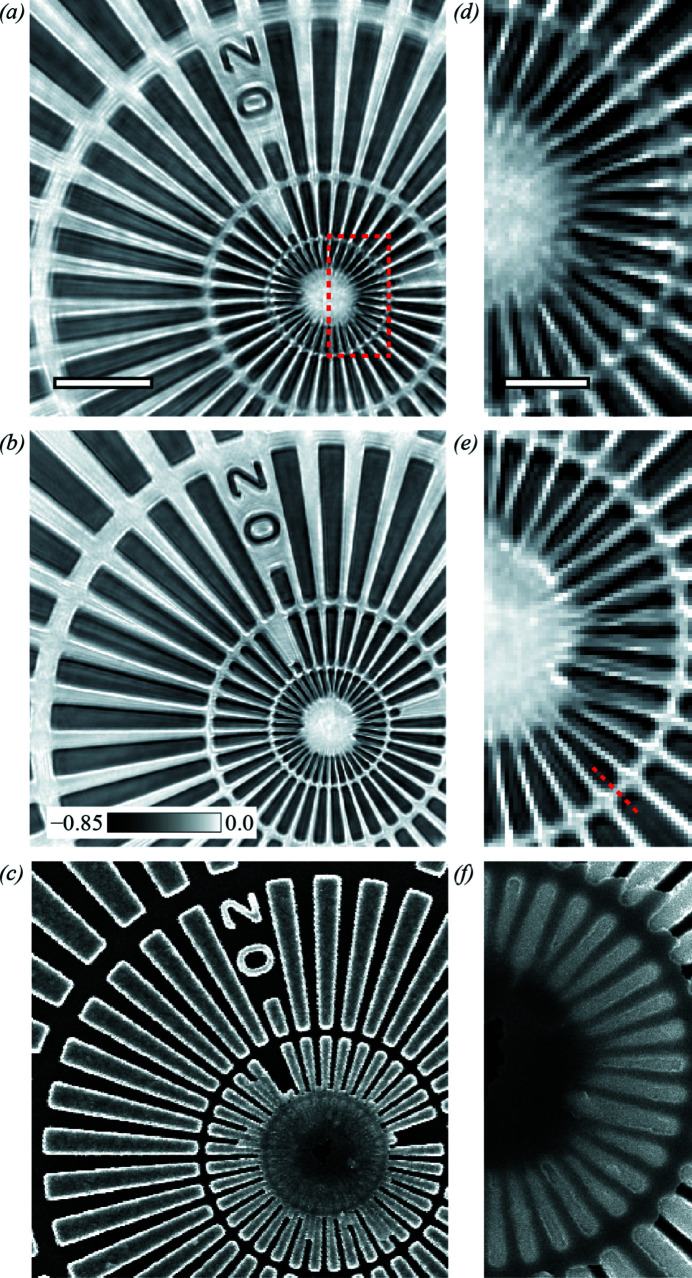
SXDM phase-contrast images of a test pattern for (*a*) externally triggered (ET) and (*b*) free-run (FR) data collection with interpolated positions. (*c*) An SEM image of the centre of the test pattern. Insets (*d*)–(*f*) are taken from the area depicted by the red box in panel (*a*), showing that FR data collection allows higher quality reconstruction of the finest details in the test object. The scale bars in panels (*a*) and (*d*) correspond to 2 µm and 500 nm, respectively, and the grey scale in panel (*b*) indicates the quantitative phase delay in radians.

SXDM reconstructions from each data collection mode are shown in Fig. 3 ▸. The ET scan shown in Fig. 3 ▸(*a*) has more artefacts than the FR scan shown in Fig. 3 ▸(*b*), most likely due to inaccurate estimation of the interpolated frame positions where there is missing data. For comparison, a scanning electron micrograph (SEM) of the sample is shown in Fig. 3 ▸(*c*), revealing that the lithography microfabrication is imperfect in the centre of the pattern, leaving an over-layer of Cr covering the central part and reducing contrast. Magnifying the image sub-region shown as a dashed rectangular ROI in Fig. 3 ▸(*a*) reveals that ET Fig. 3 ▸(*d*) resolves the finest details less clearly than FR Fig. 3 ▸(*e*).

**Figure 4:**
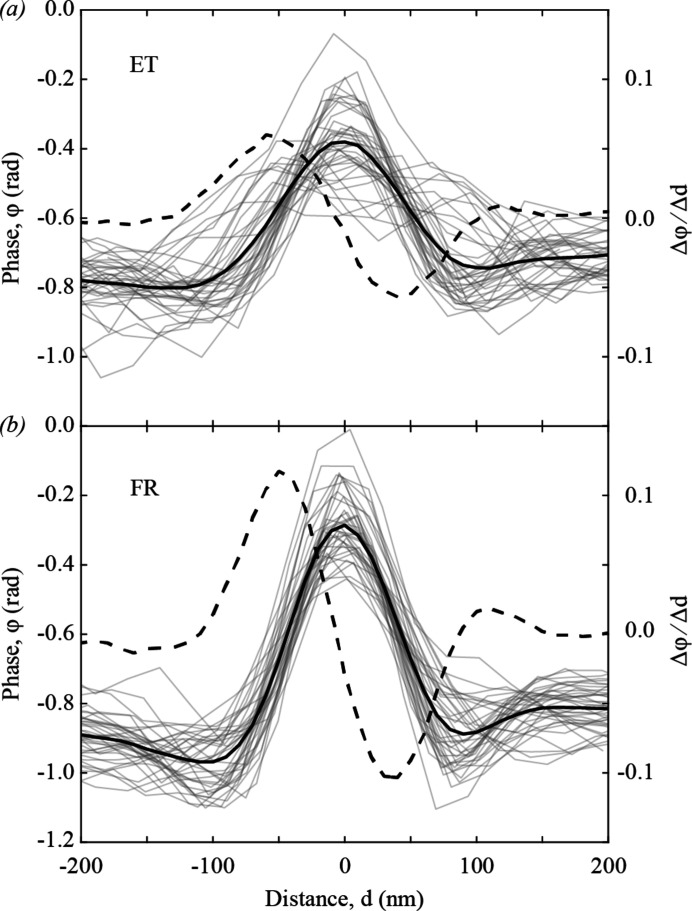
Line profiles (light-grey lines) across all 37 spokes of the test pattern from the central ring to the next. The average line profiles (black lines) and their first derivatives (dashed lines) are shown for (*a*) externally triggered (ET) and (*b*) free-run (FR) data collection with interpolated positions.

To estimate the reconstruction quality, 37 line profiles were extracted along all spokes of the test pattern, from the central ring to the next across two edges. An example profile is shown as a dashed red line in Fig. 3 ▸(*e*). The extracted profiles are shown as light-grey lines in Fig. 4 ▸. The resolution was calculated as the average FWHM across the two edges obtained from the first derivative of the average profile (Dzhigaev *et al.*, 2016 ▸), yielding 82 ± 4 nm for ET and 70 ± 1 nm for FR data collection with interpolated positions. The improved resolution obtained from FR data collection can be attributed both to measuring every frame, resulting in an increased number of photons collected per unit scan area, and to having more accurate positions for all frames, resulting in fewer reconstruction artefacts.

**Figure 5:**
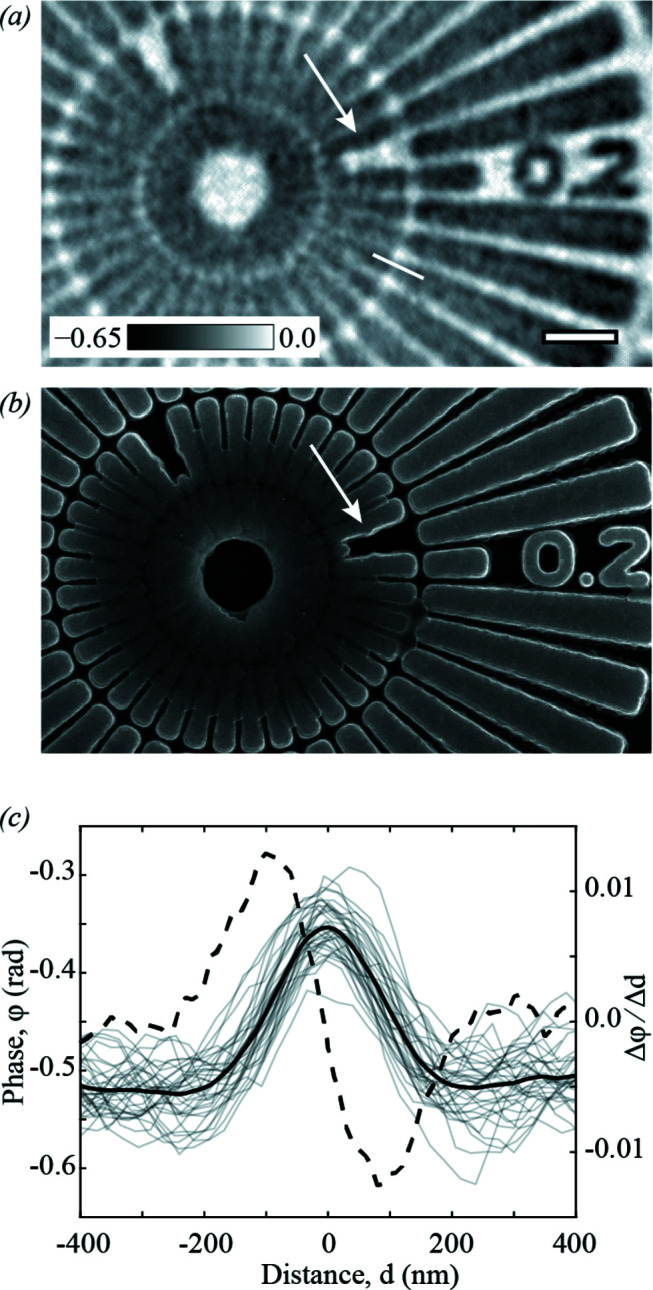
Free-run (FR) enables reliable high-speed data collection. (*a*) An SXDM phase-contrast image from FR data collected at 2500 Hz and a scan speed of 250 µm s^−1^, showing good agreement with (*b*) an SEM image of the object. Arrows in panels (*a*) and (*b*) highlight nanoscale features that can be recognized in both images. (*c*) Line profiles (light-grey lines) across 35 spokes of the test pattern from the second ring to the third. The average profile (black line) and its first derivative (dashed line) are shown. The scale bar in panel (*a*) corresponds to 1 µm and the greyscale indicates the quantitative phase delay in radians.

Fig. 5 ▸(*a*) shows the results of FR data at high scan speed and high frame rate, 250 µm s^−1^ and 2500 Hz, respectively, on a second test pattern of similar composition. The reconstructed SXDM image is faithful to the SEM image presented in Fig. 5 ▸(*b*). The reconstructed image quality was assessed in the same way as above. Line profiles were extracted across 35 spokes in the test pattern; two spokes with gaps were omitted. An example profile is shown as a solid line in Fig. 5 ▸(*a*). The extracted profiles are shown as light-grey lines in Fig. 5 ▸(*c*). The average FWHM of the first derivative (dashed line) of the average profile (solid line) in Fig. 5 ▸(*c*) yielded a resolution of 157 ± 2 nm. Comparing these results with Fig. 3 ▸, we note that the reduced flux, combined with the increased scan line spacing to 500 nm, results in three orders of magnitude fewer photons per square micrometre of scan area. The achievable resolution could therefore be expected to degrade by a factor of up to 6.5, according to estimations of dose-resolution limits in diffraction microscopy (Howells *et al.*, 2009 ▸). This reasoning would imply that the resolution in Fig. 3 ▸(*b*) could be improved by increasing *M*. However, reconstructing the data in Fig. 3 ▸(*b*) with *M* = 472, by extending the array to the edge of the detector, yields SXDM images (not shown) with 13.3 nm pixels and spatial resolution of 66 ± 2 nm. Based on the scattered intensity statistics in the data we conclude that the resolution-limiting factor is not the detection solid angle. Instead, we attribute this result to several other experimental factors, including the sample motion due to fly scanning, which may only be partially accounted for by the modal reconstruction, the beam stability during the scan and the positioning uncertainty resulting from encoder readout with a precision of 20 nm. We note that including the turnaround area in the ptychographic reconstruction introduced no detrimental effect on reconstructions.

**Figure 6:**
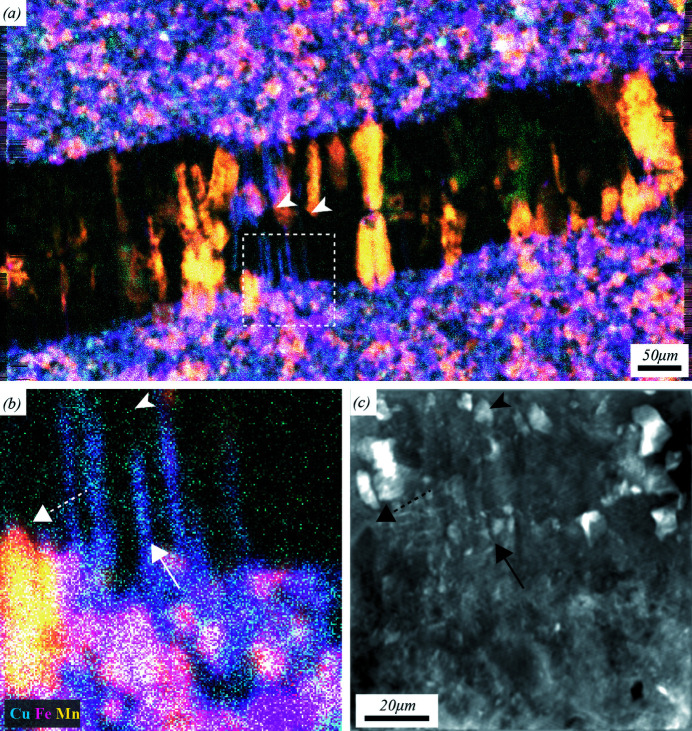
A large-area correlated SXDM-XFM image of a small vein in a freestanding thin section of shale. (*a*) An XFM image of the entire 800 µm × 440 µm scan area is shown in cyan, magenta and yellow for Cu, Fe and Mn, respectively. (*b*) An enlargement of the region of interest (ROI) of the 100 µm × 100 µm area indicated by the dashed square in panel (*a*). (*c*) An SXDM phase-contrast reconstruction in the same ROI, revealing high-resolution high-contrast structural information. For explanation, see main text.

Large-area XFM of thin geological sections provides a multitude of quantitative information, over orders of magnitude in length scales, which is important for answering questions at the forefront of geological sciences (Akker *et al.*, 2021 ▸). Fig. 6 ▸(*a*) shows an 800 µm × 440 µm XFM map of a shale sample with a ∼175 µm wide vein filled with quartz and calcite grains as a tricolour overlay, with Cu in cyan, Fe in magenta and Mn in yellow. The pixel size in this image is 500 nm, although the spatial resolution is limited by the probe size at the sample of around 2.0 µm FWHM. Fig. 6 ▸(*b*) shows a 100 µm × 100 µm ROI of this XFM image, while Fig. 6 ▸(*c*) shows the SXDM reconstruction of the coherent diffraction data in the same ROI, wherein the reconstructed pixel size is 24.5 nm. SXDM phase images clearly show details not observed in the XFM images. The white stemless arrowheads in Fig. 6 ▸(*a*) mark the typical pyramidal endings of euhedral quartz crystals (which appear black) growing into the vein. In the enlarged XFM map [Fig. 6 ▸(*b*)], the region near the tip of such a quartz crystal does not show any internal elemental zoning (white stemless arrowhead) while the SXDM map [Fig. 6 ▸(*c*)] unveils significant internal structure (black stemless arrowhead), possibly reflecting different growth increments. The solid white arrow in Fig. 6 ▸(*b*) marks a linear region of chemically homogeneous chlorite overgrowth on a quartz grain boundary. The corresponding region in the SXDM map [Fig. 6 ▸(*c*), solid black arrow] resolves the subvertical interfaces of the chlorite–quartz interfaces and displays a rich internal structure where the quartz crystal locks onto the shale matrix. The dashed arrows in Figs. 6 ▸(*b*) and 6 ▸(*c*) show how clearly the tip of a euhedral dolomite crystal is resolved in SXDM compared with XFM. It is clear that the combination of SXDM phase and XFM elemental maps delivers insights not available from either method in isolation.

## 6. Conclusions

We have implemented and demonstrated high-speed reliable free-run SXDM on the Australian Synchrotron XFM beamline, including a data analysis pipeline that permits general user access to ptychographic imaging. By allowing the data to be collected by the detector without sending an individual trigger to the detector for each exposure, and interpolating stage events recorded on a far finer grid than the scan positions, robust high-quality SXDM reconstructions are obtained and non-measurement dead time is eliminated. This ensures that all possible photons incident on the sample are collected and the data reliably reconstruct to high fidelity, even for sample velocities up to 250 µm s^−1^, and at a data collection rate of 140 µm^2^ s^−1^, which to our knowledge is the fastest X-ray ptychography reported to date. We have demonstrated that this provides ultrastructural phase-contrast context for XFM images at approximately 13 times finer resolution than the beam size over large areas. The resolution can be further increased by slower scanning. However, we found that the resolution limit is dictated by experimental factors including the fly-scan motion of the sample during each diffraction measurement, the beam stability during a scan and the positioning accuracy, rather than the diffracted intensity measured per frame.

We anticipate that fast-scanning simultaneous XFM and SXDM over large areas will have an immediate and powerful impact in materials science, advanced manufacturing, and particularly in geoscience, where structure and chemistry are inherently linked over a large range of length scales. Moreover, the ptychographic reconstruction of a high-resolution complex probe beam could yield resolution enhancement of the XFM images through deconvolution (Vine *et al.*, 2012 ▸). This method is relatively detector independent, applicable to most fast-readout low dead time systems. As detector count-rate capabilities and coherent flux improve, we anticipate that scan speeds such as this will become routine, and ptychography data collection rates will become compatible with fast-scanning X-ray fluorescence microprobes and nanoprobes such as the future Australian Synchrotron Nanoprobe beamline.

Upcoming milestones in the SXDM development roadmap on the XFM beamline are the installation of a DMM providing 20–30× flux increase, permitting broadband SXDM (Enders *et al.*, 2014 ▸; Yao *et al.*, 2021 ▸), and the implementation of a high-precision rotation stage for ptychographic X-ray computed tomography (Dierolf *et al.*, 2010 ▸).
